# Supplementary figures and images for: The transsulfuration pathway suppresses the embryonic lethal phenotype of glutathione reductase mutants in Caenorhabditis elegans
Source: G3 (Bethesda). 2025 May 7;15(8):jkaf102. doi: 10.1093/g3journal/jkaf102 (PMC12341920; doi:10.1093/g3journal/jkaf102)

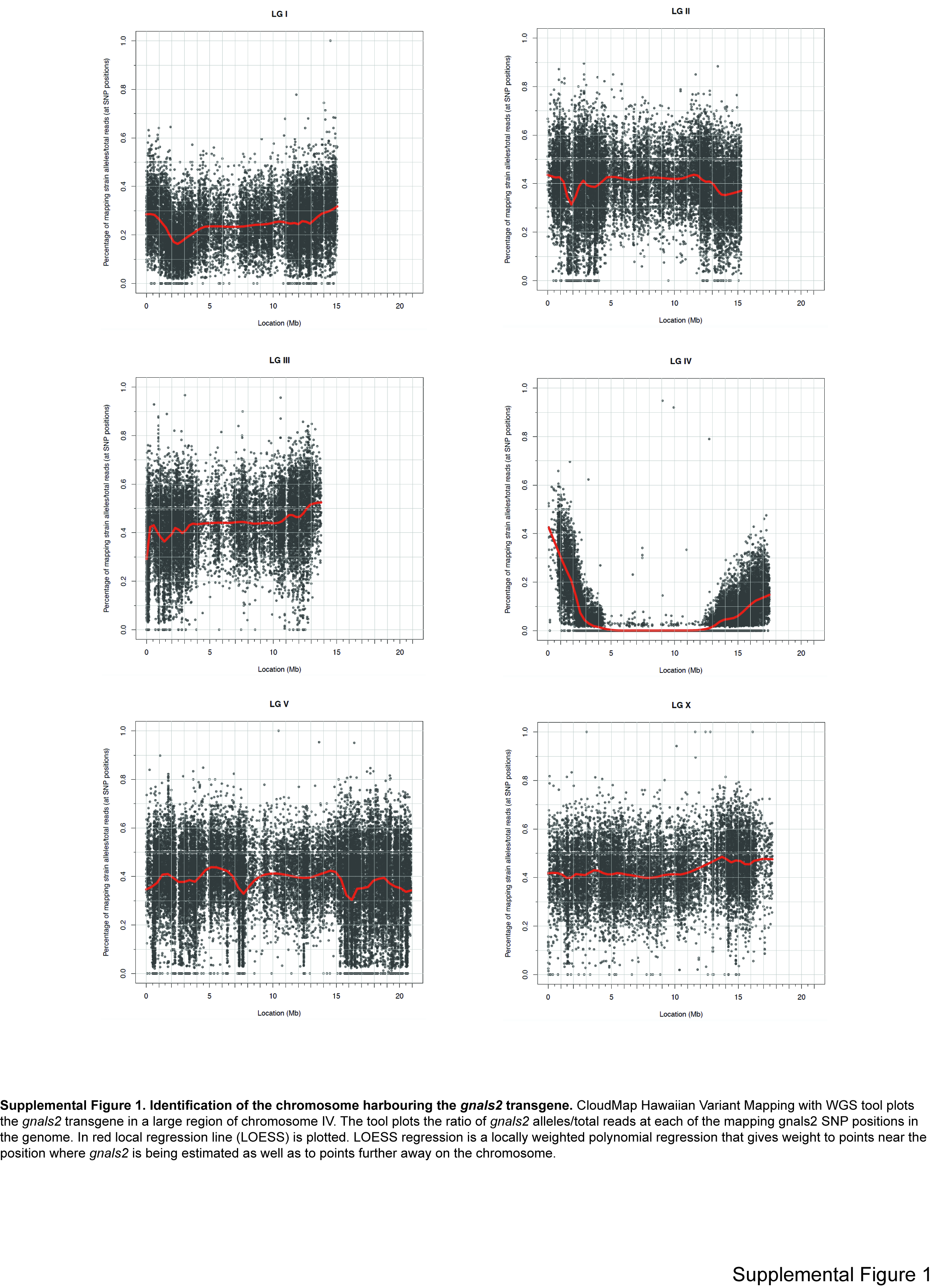

Supplement: jkaf102_Supplementary_Data [file jkaf102_supplementary_data.zip › Supplemental_Figure_1_G3-2025-405727.tif]

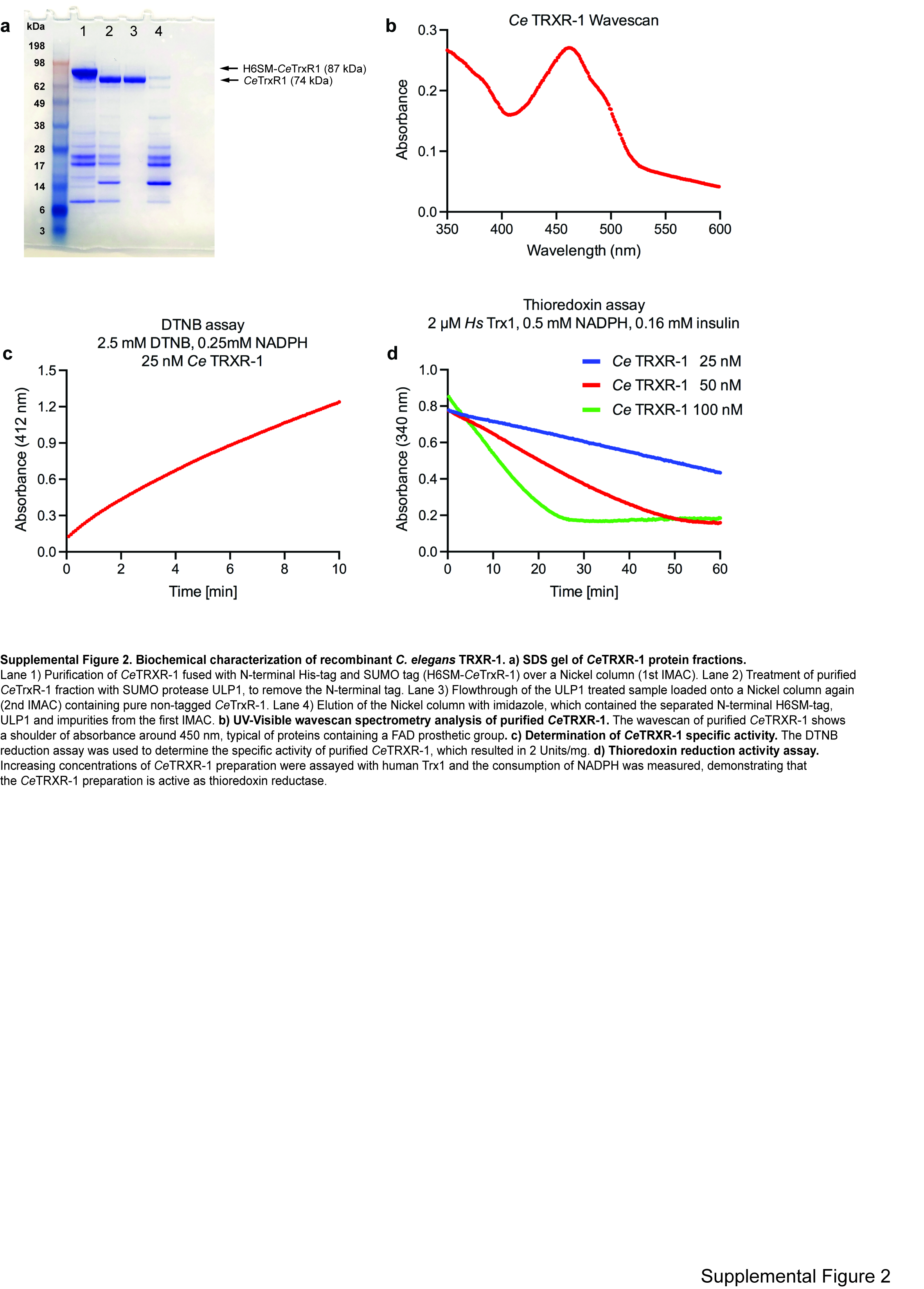

Supplement: jkaf102_Supplementary_Data [file jkaf102_supplementary_data.zip › Supplemental_Figure_2_G3-2025-405727.tif]

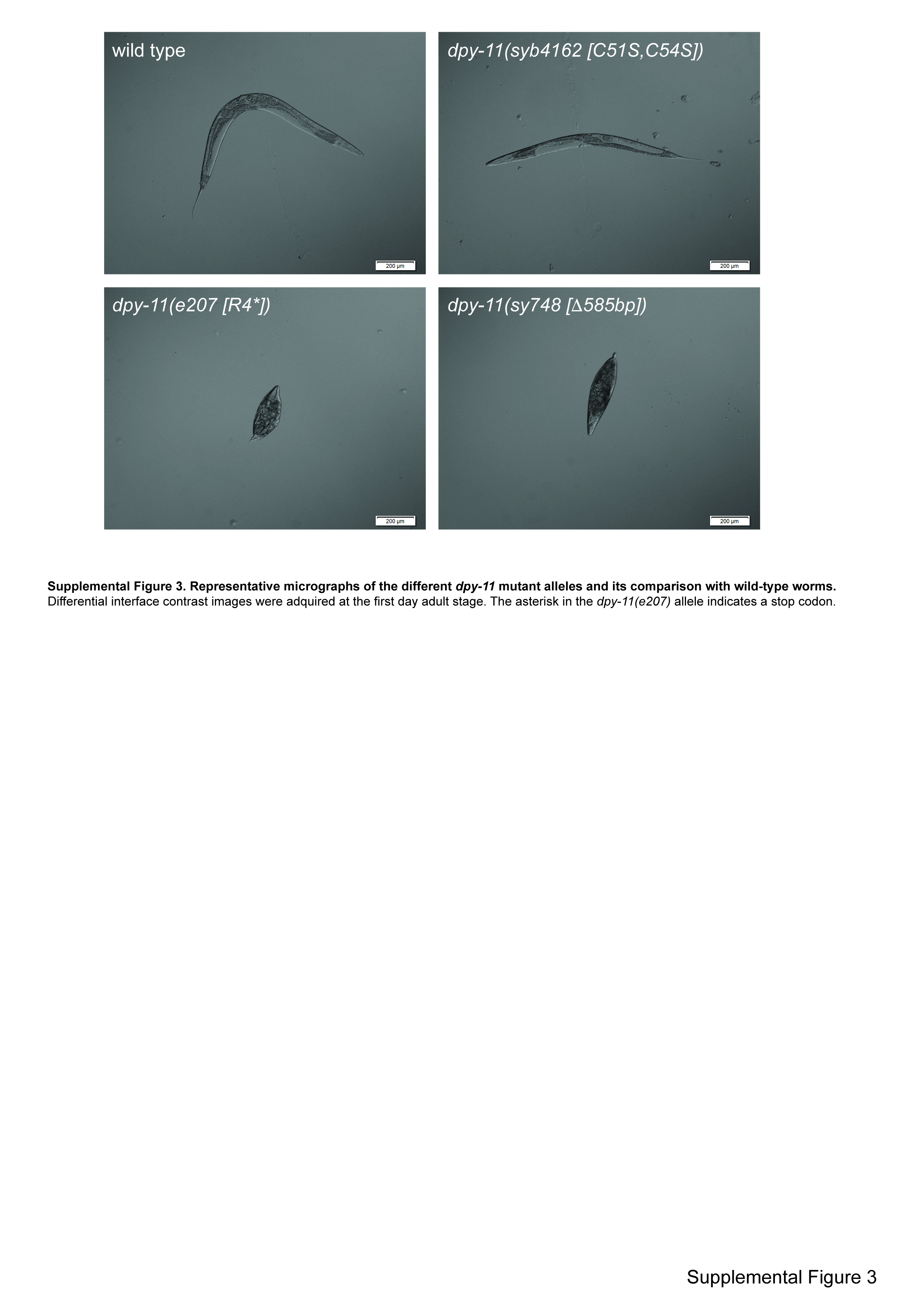

Supplement: jkaf102_Supplementary_Data [file jkaf102_supplementary_data.zip › Supplemental_Figure_3_G3-2025-405727.tif]

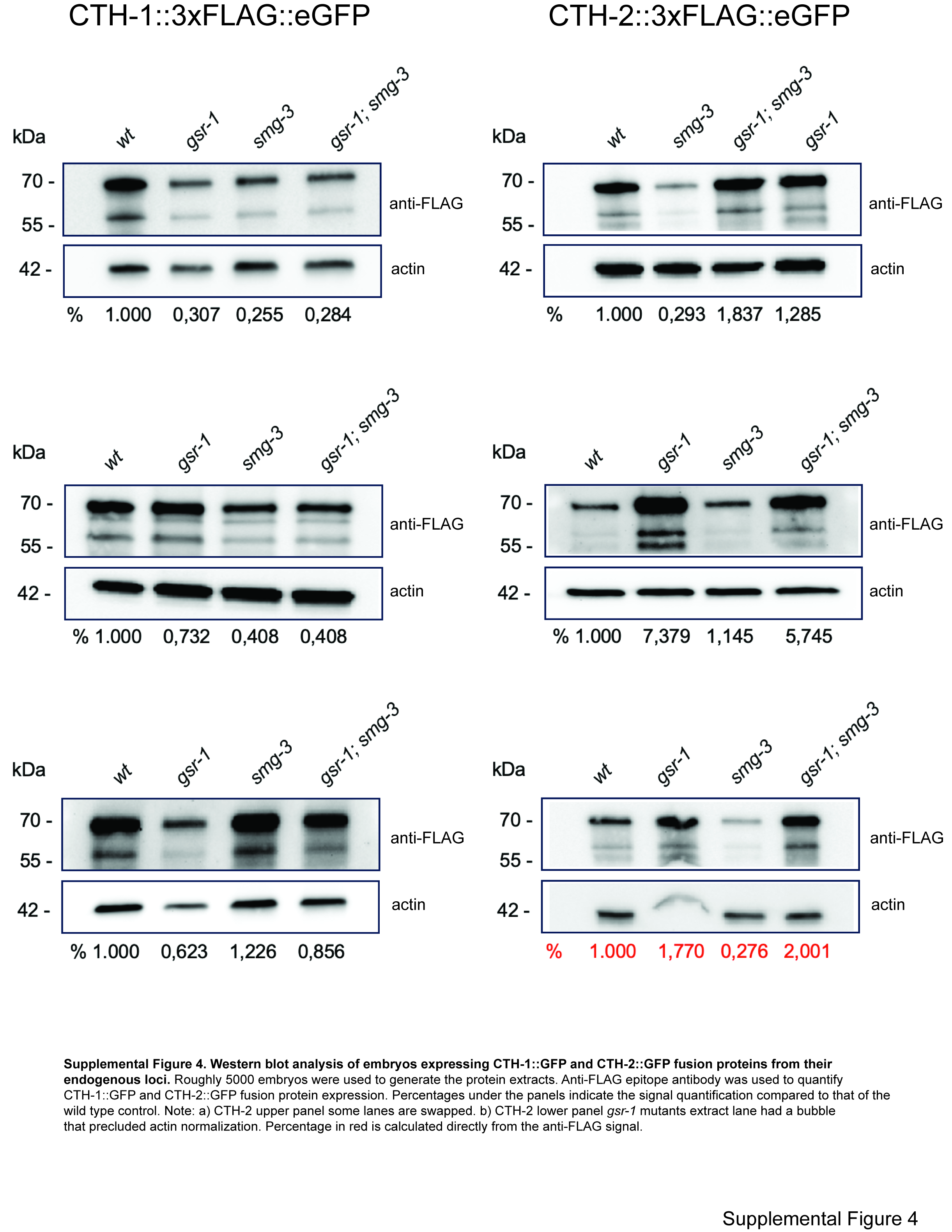

Supplement: jkaf102_Supplementary_Data [file jkaf102_supplementary_data.zip › Supplemental_Figure_4_G3-2025-405727.tif]

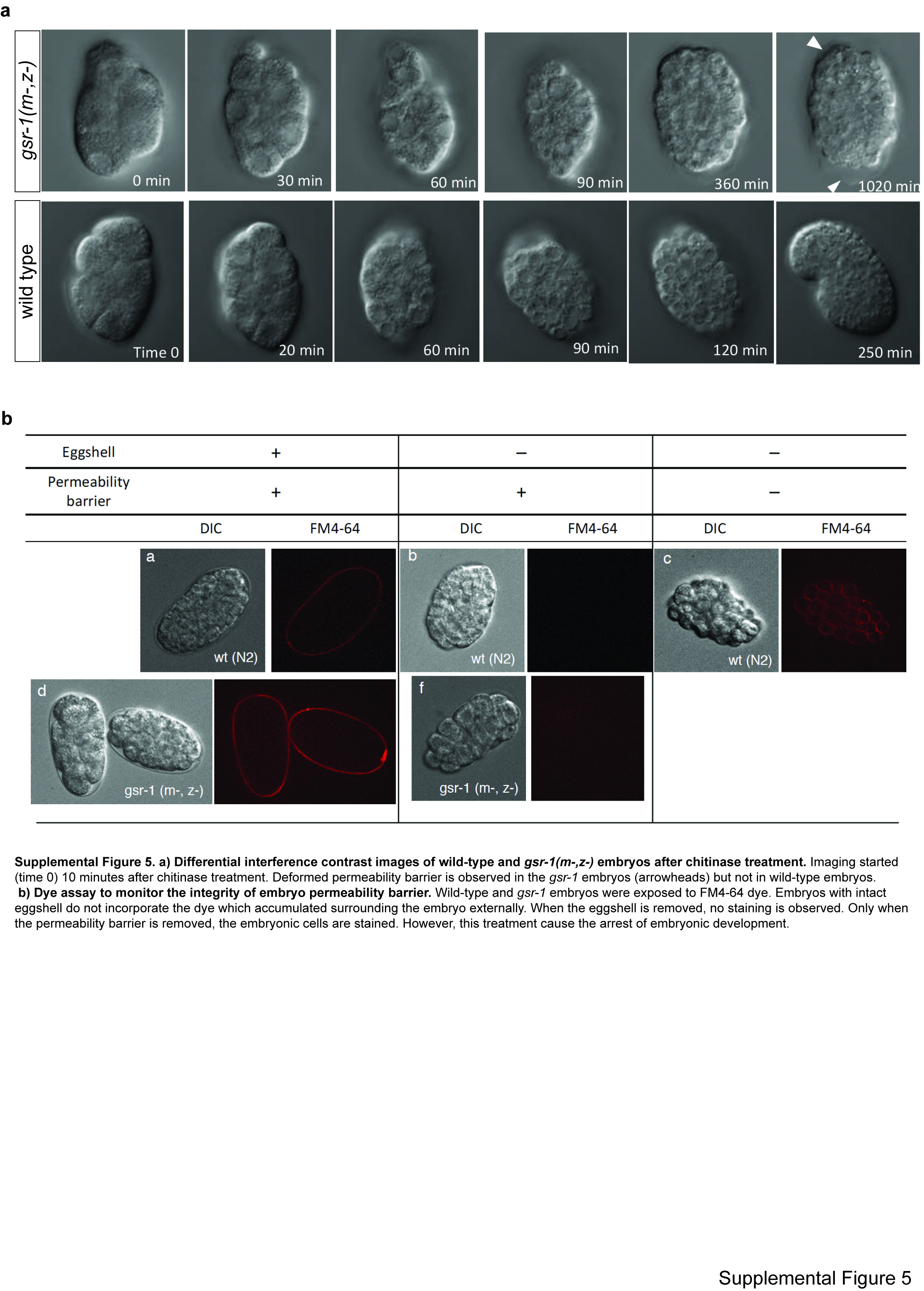

Supplement: jkaf102_Supplementary_Data [file jkaf102_supplementary_data.zip › Supplemental_Figure_5_G3-2025-405727.tif]
